# Decentralising and integrating HIV services in community-based health systems: a qualitative study of perceptions at macro, meso and micro levels of the health system

**DOI:** 10.1136/bmjgh-2016-000107

**Published:** 2017-01-20

**Authors:** Lilian Otiso, Rosalind McCollum, Maryline Mireku, Robinson Karuga, Korrie de Koning, Miriam Taegtmeyer

**Affiliations:** 1 LVCT Health, Nairobi, Kenya; 2 Department of International Public Health, Liverpool School of Tropical Medicine, Liverpool, UK; 3 Royal Tropical Institute, Amsterdam, The Netherlands

## Abstract

**Introduction:**

HIV services at the community level in Kenya are currently delivered largely through vertical programmes. The funding for these programmes is declining at the same time as the tasks of delivering HIV services are being shifted to the community. While integrating HIV into existing community health services creates a platform for increasing coverage, normalising HIV and making services more sustainable in high-prevalence settings, little is known about the feasibility of moving to a more integrated approach or about how acceptable such a move would be to the affected parties.

**Methods:**

We used qualitative methods to explore perceptions of integrating HIV services in two counties in Kenya, interviewing national and county policymakers, county-level implementers and community-level actors. Data were recorded digitally, translated, transcribed and coded in NVivo10 prior to a framework analysis.

**Results:**

We found that a range of HIV-related roles such as counselling, testing, linkage, adherence support and home-based care were already being performed in the community in an ad hoc manner. However, respondents expressed a desire for a more coordinated approach and for decentralising the integration of HIV services to the community level as parallel programming had resulted in gaps in HIV service and planning. In particular, integrating home-based testing and counselling within government community health structures was considered timely.

**Conclusions:**

Integration can normalise HIV testing in Kenyan communities, integrate lay counsellors into the health system and address community desires for a household-led approach.

**Key questions**

**What is already known about this topic?**

- In many settings HIV services at the community level are provided through vertical programmes whose sustainability is under threat. Studies on decentralising HIV services in Sub-Saharan Africa have demonstrated that while using an integrated service delivery approach is feasible and effective, questions remain about whether and how this can be implemented within a holistic community health platform.

**What are the new findings?**

- This is the first study in Kenya to comprehensively explore the perspectives of a range of stakeholders on the acceptability and feasibility of integrating HIV services into existing community health structures. We found widespread support for integrating HIV service at the community level.

**Recommendations for policy**

- Integrating HIV services such as testing and counselling, linkage and adherence support with community health programmes could prove to be a sustainable approach to scaling-up and normalising community HIV services. This task-shifting approach, however, needs to build on existing frameworks in order to strengthen—and not distort—community health systems.

## Introduction

Many African countries are adopting the UNAIDS 90–90–90 target of ensuring that 90% of people living with HIV know their status, 90% of the people with diagnosed HIV infection receive antiretroviral therapy and 90% of those on treatment have viral suppression.^1^ Reaching these targets requires a substantial increase in service coverage but is simply impossible to achieve through traditional facility-based HIV services. Community-based approaches have been shown to increase HIV testing uptake, increase the proportion of first-time testers, identify persons earlier in the course of HIV-infection,^2–4^ and improve linkage to care.^5–7^ Currently, data on the effectiveness of these community-based approaches are derived largely from donor-funded projects delivered through parallel vertical programmes. For example in Kenya, where our study was conducted, about 38% of HIV testing and counselling has been carried out in the community by lay counsellors salaried on specific projects.^8^ These lay counsellors do not have a clinical background, they are trained only to conduct testing and counselling, are employed by non-governmental organisations and are not a recognised cadre in Kenya's Ministry of Health scheme of service.^9^

Despite evidence of being effective, donor funding for community HIV services in Sub-Saharan Africa is flat-lining or decreasing,^10^ with calls for integrating HIV services into community health as a strategy to reduce stigma and sustainably increase HIV services.^11^ ^12^ However, integration may not be as straightforward as it seems; a recent review of integrating sexual health services into primary care revealed a number of challenges in coordination, logistics, human resources (HR), training, supervision and financing.^13^

**Figure 1:**
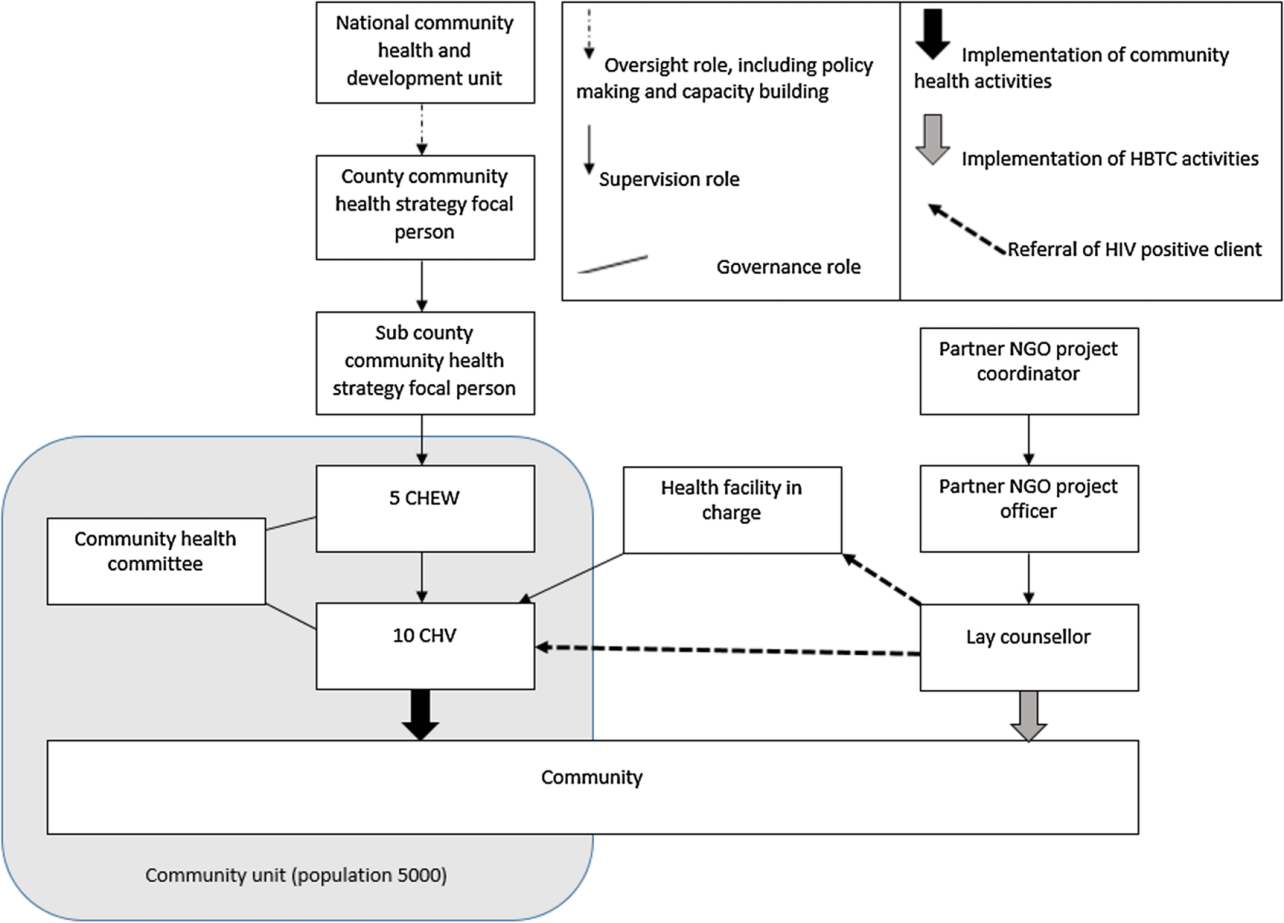
Revised community health structures for government workers and lay counsellors. CHEW, community health extension worker; HBTC, home-based testing and counselling; NGO, non-governmental organisation.

At the same time as donor funding declines in Kenya, two changes in health provision offer a window of opportunity for integration. First, Kenyan health services have devolved from a single central government to 47 county governments with decisions around service delivery priorities now being determined and funded at the county level,^14^ ^15^ and second, the Kenyan community health strategy is being revised.^16^ Figure 1 depicts government community health structures according to the revised community health strategy (2014), including how lay counsellors work in parallel but with referral links to government health structures and services. The strategy defines community health volunteer roles as raising awareness, promoting early service seeking behaviour, defaulter tracing and caring for the chronically ill.^16^ Community health extension workers are employed by the government to provide support and supervision to community health volunteers and to provide diagnosis and treatment such as for malaria and other childhood illnesses, but they have no specific HIV-related tasks.^16^

Our aim of this study was to provide timely information on possibilities for integrating HIV services at this critical juncture in Kenya. We set out to describe perceptions of current policy and practice for HIV service delivery at the community level and to explore opportunities to integrate HIV services among the key community health actors at the various levels of the Kenyan community health system. Box 1 presents key definitions in relation to community HIV services.

Box 1 **Key study definitions in relation to community HIV services**

▸ Community health worker (CHW): Any individual delivering healthcare, trained in the skills needed for the intervention but with no certificate or degree in tertiary education. In Kenya this term includes both CHVs and CHEWs.

▸ Community health volunteer (CHV): A volunteer CHW trained in a government-approved curriculum, who is responsible for 20 households, offering advice on disease prevention and control, providing family and maternal health services, promoting environmental health and sanitation, and performing basic curative tasks

▸ Community health extension worker (CHEW): A trained health worker employed by the Kenyan government in a link health facility, providing support and supervision to CHVs

▸ Lay counsellor: An individual who has completed secondary education and been trained specifically to conduct HIV testing and counselling; usually employed by an NGO.

▸ Community HIV services: Services provided in the community including home-based HIV counselling and testing, linkage for care and treatment, and home-based care.

▸ Integration: This refers to the incorporation of community HIV services traditionally carried out by vertical programmes into the existing Ministry of Health community health structures.

## Methods

We used qualitative methods to explore the perceptions of actors at the macro level (referred to as policymaker level), the meso level (county managers implementing services) and the micro level (community health extension workers, community health volunteers, lay counsellors and community members). This macro-meso-micro framework has been used in studies of policy implementation and draws on existing theory that participants at each level ‘frame’ their understanding differently.^17–22^ We used a range of interview types shown in table 1. With policymakers and health managers we used in-depth interviews to gain insights into their perspectives. We also interviewed home-based testing and counselling clients individually to explore sensitive issues in private. These interviews were supplemented by anonymous online semistructured questionnaires with lay counsellors—a method selected to avoid potential biases resulting from individuals being interviewed by researchers employed in their own organisation. Finally, we used focus-group discussions with community health volunteers and community members as group interaction can generate ideas and conversations that enable understanding of similar and diverging views within a group.^23^ ^24^

**Table 1:** Characteristics of study respondents

	National	Nairobi	Kitui
In-depth interview respondents			* *
Policymakers	3 male 1 female	0	0
Subcounty managers	0	0 male 3 female	3 male 0 female
Facility in-charges	0	0 male 2 female	1 male 1 female
Community health extension workers	0	4 male 4 female	4 male 4 female
Home-based testing and counselling clients	0	0 male 5 female	1 male 4 female
Total (n=40)	4	18	18
Characteristics of semistructured questionnaire respondents			
Lay counsellors		6 male 7 female	3 male 9 female
Total (n=25)		13	12
Characteristics of focus-group discussion respondents			
Community members (2 FGDs per county)		5 male 15 female	10 male 12 female
Community health volunteers (3 FGDs per county)		12 male 24 female	11 male 25 female
Total (n=114)		56	58

FGDs, focus group discussions.

*Study sites**:***The study was conducted in 2013 in two counties in Kenya selected for their geographical and social variation and recent exposure to community HIV services in the form of home-based HIV testing and counselling. Kitui, a rural county located in southeast Kenya, has an estimated population of 1 065 329 with poor health services and outcome indicators, high rates of childhood malnutrition and an HIV prevalence rate of 4.3%.^25^ ^26^ Nairobi is a densely populated urban county with 3.7 million people, numerous slum areas, inequitable health service usage and an HIV prevalence of 8%.^25–27^ We selected community units in three of six subcounties in Kitui and four of nine subcounties of Nairobi on the basis of their having received home-based testing and counselling services from lay counsellors within the previous 2 years.

We selected participants purposively and continued interviewing until saturation was reached (see table 1). We selected all policymakers and subcounty managers directly involved in decision-making on community health services. We interviewed facility in-charges and community health extension workers (CHEWs) in the associated link facilities and asked all lay counsellors from the non-governmental (NGO) employees who had offered services in the study areas to complete the questionnaire. Community health volunteers (CHVs) and general community members were convenience sampled with the help of local administrators and focus group discussions (FGDs) were conducted on an ‘all welcome basis’. Male and female home-based HIV testing clients were approached in the communities by CHVs who had been involved in community mobilisation. Our survey with lay counsellors had 100% uptake.

*Data collection**:*** Topic guides for FGDs explored a broad range of priorities and concerns at the community level and included questions on the types of services available in the community, perceptions of the quality of services, potential roles of lay counsellors and CHVs and what they thought about integrating HIV services. We included some similar questions across methods to ensure triangulation and comparison. The survey with lay counsellors specifically sought to determine how they link with the community, their knowledge of other community duties and willingness to take on more community roles. Topic guides were developed, translated into Kiswahili, back translated to English and piloted prior to use. Trained interviewers facilitated all discussions. Data from in-depth interviews and focus-group discussions were digitally recorded and transcriptions counterchecked with audio files. Regular meetings were held between interviewers and senior researchers to review emerging themes, and a coding framework was developed jointly through an iterative process starting with the major themes covered in the topic guides.^23^ Findings were presented to county stakeholders as well as members of the national community health operations research technical working group, and feedback from stakeholders was integrated into the analysis through alterations to the coding framework and final analysis. The study protocol was approved by the Kenya Medical Research Institute Ethics and Review Committee and the Royal Tropical Institute, Amsterdam (KIT) Research Ethics Committee.

## Results

Themes emerged in three key areas: (1) current and potential HIV testing services at the community level; (2) the issues and challenges around vertical programmes; (3) the perceptions of HIV service integration at community level. For each area we present and examine commonly held views across the range of participants at the policymaker, implementation and community levels.

### HIV services delivery at the community level: policy, practice and potential for integration

Our findings revealed that community-level actors (primarily community health volunteers) do interpret their general roles as outlined in the official policies as applying to providing community HIV services. These included health promotion “(CHVs) usually visit us at home, and they tell us how we can protect ourselves from HIV”. (Kitui Community 1); referral of pregnant women for prevention of mother-to-child transmission (PMTCT) “If a woman is HIV positive and they are pregnant you keep reminding them to go and give birth in the hospital so that the baby does not get the virus”. (Kitui CHV 1); defaulter tracing for antiretroviral therapy, PMTCT and tuberculosis (TB) treatment (more commonly described in Nairobi than Kitui county) “If the doctor finds there is a defaulter, they will ask the CHV to follow up and know what is wrong”. (Kitui CHV 1).

In addition to the commonly described roles, a minority of respondents also described other roles including condom distribution; referral and linkage of HIV-positive clients to existing government HIV services; assisting with couples' disclosure; referral of patients with signs and symptoms of TB; and home-based care for HIV-positive clients. “Clients who test HIV positive are linked to community health workers with a mandate to ensure that they access care” (HBTC Counsellor). All of the roles identified (including those commonly and less commonly described) align with the Kenya Community Health Strategy (2006) and are summarised in table 2 below.

**Table 2:** HIV roles for community health volunteers: policy, practice and opportunities for integration identified by respondents

Area of focus in the community strategy*	HIV-related tasks described in community health policy†	HIV-related tasks described by meso-level and micro-level respondents	Additional HIV-related tasks described by CHVs (micro-level)	Suggestions made by all levels of respondents for potential roles for lay counsellors, CHVs and CHEWs in an integrated approach to HIV services
Disease prevention and control to reduce morbidity, disability and mortality	Raise awareness on disease causation, control and prevention, in particular STI/HIV/AIDS	HIV prevention education	Condom distribution	Community level respondents suggest that CHVs and CHEWs continue providing HIV prevention education and explore opportunities to expand CHV distribution of condoms. They feel lay counsellors were acceptable to their clients who described seeking them out in the event of any problems. Lay counsellors identified roles which they could take on in addition to their current HBTC roles.
Family health services to expand family planning, maternal, child and youth services	Promote early service-seeking behaviour	Referral of pregnant women for prevention of mother-to-child transmission (PMTCT) and hospital deliveries	Referral and linkage of HIV positive to care currently is carried out by NGO-supported lay counsellors	Policymakers felt home-based HIV testing could be conducted by CHEWs if appropriately trained, with referral and linkage to care (as currently carried out by NGO-supported lay counsellors). CHVs expressed a desire to be trained to conduct HIV testing and felt it would help extend coverage of testing services, particularly among youth.
Information education communication (IEC) for community health promotion and disease prevention	Sensitise, mobilise and organise community to ensure leadership and awareness of rights and responsibilities in health	Mobilisation and referral for HBTC	Aid in couples disclosure	Respondents at macro and meso levels expressed the need for greater community engagement around HIV issues, opportunities for assisting with couples’ disclosure and for normalisation of HIV through HBTC
Disease control Community-based referral system	Conduct community directly observed treatment (C-DOTS) and defaulter tracing	Defaulter tracing for antiretroviral therapy (ART), PMTCT and TB medication	Referral of patients with signs and symptoms of TB	Policymaker respondents (macro level) see the potential for community-based HIV management through a decentralised approach
	Care for chronically ill	None	Home-based care for HIV-positive community members	CHEWs and CHVs see an opportunity for holistic care and expanding home-based care

*Strategic Plan of Kenya Taking the Kenya Essential Package for Health to the Community.

†A Strategy for the Delivery of Level One Services, 2006 pages 10–13.

CHEWs, community health extension workers; CHVs, community health volunteers; HBTC, home-based testing and counselling; NGOs, non-governmental organisations; STI, sexually transmitted infections; TB, tuberculosis.

Community-health extension workers, community health volunteers and lay counsellors all expressed willingness to take on additional professional roles. Lay counsellors were thought by policymakers to be able to take on community health extension workers tasks in relation to community health, and counsellors endorsed this in their own responses. It was also commonly accepted that community health extension workers could take additional roles for home-based testing and counselling if provided with the necessary training. This was echoed by some community health volunteers who wanted to receive home-based testing and counselling training in response to community demand for those services.

So that when we are attending to this client, we attend to all issues of nutrition, home-based care issues, issues of TB, like that, so that when I come I come fully, not I come, then another person comes for TB, then another person comes, I just want to go and do everything… because these people in the community need care, they need people, who can follow them up. (NBO CHEW 08)

### Challenges of vertical programming at the policymaker level play out at the county and community level

Participants from all levels were aware of the multiple HIV-related tasks at the community level, noting that they were often driven by vertical programmes in response to local need or external funding.

HBTC has been run vertically in this country … (National Policymaker 3)

Challenges in coordination, coverage, duplication and lack of clarity on roles were highlighted as well as concerns that HIV services were a major driver of such issues. Policymakers and subcounty managers highlighted that the absence of government-funded community health services in some areas resulted in a vacuum leading to NGO-driven scale-up and resultant inequities in coverage and focus—with NGOs providing disease-specific (often HIV-specific) services and selecting geographical areas convenient to their organisation. One national policymaker reflected a common opinion that the high level of vertical programming for both community health services and HIV at the national level had a harmful impact on overall service delivery at the community level:

At the top there, the structures are parallel, when it is at the top there, it is one problem, but when it gets back to the community becomes a big problem. (National Policymaker 2)

At the implementation level respondents highlighted that system integration required a variety of county structures and processes to be put in place, with a focus on communication between the levels, coordination among stakeholders and robust systems for supply chains and referral. There was an identified need to improve interorganisational relationships between stakeholders at the community level. In Nairobi, a member of the subcounty health management team described poor communication between the subcounty health management team and NGOs. There was also variation between HIV services provided by NGOs, with some NGOs providing a focus on services for children who are HIV positive, others concentrating on HIV-positive pregnant women and others on the provision of home-based testing and counselling, all with patchy coverage. In addition to this there were differences described in the remuneration of community health volunteers at the community level within and beyond HIV services:

We sent out a circular that they (all community health volunteers) should be remunerated…but most partners are inclined towards HIV, hence those CHVs inclined to HIV are the ones remunerated…. (National Policymaker 4)

### Perceptions of HIV service integration at the community level

There was support across all respondents for an integrated approach incorporating home-based testing and counselling within existing community health activities carried out by government co-ordinated community health actors (community health extension workers and community health volunteers). This was summarised by a national policymaker who said:

HBTC has been run vertically in this country … the only way to handle that issue is to make it integrated so that the community health extension worker becomes the person who is responsible in the HBTC. (National Policymaker 3)

Participants saw opportunities and benefits as well as challenges in integration at each level of the health system and for a range of cadres (see table 3). On the one hand, all groups of respondents identified the need for more holistic care at the community level with potential benefits perceived for the micro level. The benefits of normalising and sustaining an approach to HIV testing at the community level were identified by lay counsellors and were also noted by policymakers who raised concerns over funding flat-lining and the need for a unified cadre able to provide HIV testing as part of the package of care. On the other hand, concerns were raised about the practical feasibility of integration including issues of political backing, the need for consistent budget allocation for community health activities, strengthening supply chain structures to ensure community providers have adequate supplies and the need for recognising and investing in training and supervising community providers of HIV testing and counselling (HTC) services.

**Table 3:** Perceptions of the potential impacts of an integrated model at macro, meso and micro level

	Potential impacts on CHEWs	Potential impacts on existing lay counsellors	Potential impacts on CHVs	Potential impacts at the community level
Perceptions at macro level national and county policymakers	CHEWs trained and competent in HTC Too many tasks could dilute quality and accountability	Skilled group taken up as part of Community Health Services and ‘home testing’ within health system	Increased clarity on HBTC support functions of CHVs Concerns about workload	Normalises HIV testing Enables holistic services to be delivered at home
Perceptions at meso-level and county-level implementers	Improved county coordination of vertical programmes and of interorganisational relationships	Integrated approach to training, supervision, data management, commodities and supplies. Potential for stock outs	Improved supervision and support for HIV services offered, able to conduct current HIV tasks within an official framework	Opportunity to extend HIV services within the community
Perceptions at micro level—community level actors	CHEWs able to offer HBTC at home for pregnant women, TB patient contacts, families of HIV-positive individuals	Offer services additional to current HBTC roles	Holistic picture of the household; able to mobilise for HIV testing, support linkage from community to health facility; able to respond to community demand for HTC or to provide condoms	Availability of condoms at the community level Easier access to HTC, increased uptake especially among men Holistic care available at the community level Concerns about confidentiality and stigma

CHEWs, community health extension workers; CHVs, community health volunteers; HBTC, home-based testing and counselling; HTC, HIV testing and counselling; TB, tuberculosis.

Implementers, county staff and national policymakers raised the importance of interorganisational relationships (eg, strategic alliances and common governance mechanisms) as well as partnerships between professionals both within and between organisations.

Community members and lay counsellors raised confidentiality as a concern in a model integrating home-based testing and counselling in the community strategy, although contrasting opinions were expressed and solutions also offered as illustrated here:

They should be trained to ensure confidentiality. They can visit us, give us counselling and test us…but there must be some precautions on how they will be trained. (Kitui Community 1).

Respondents had additional suggestions such as using an alternative provider for home-based testing and counselling at the community level; using a CHV from a different community; and including confidentiality as a selection criterion for community health volunteers.

## Discussion

Our study shows that current HIV service provision at the community level already goes beyond policy with community-level HIV services that are supported by NGOs including adherence counselling, referral, defaulter tracing, home-based care and HIV testing in a responsive manner. Study respondents described a range of challenges presented by the current vertical programming and a expressed a desire for better integration. Our findings revealed support for integrating HIV services at the community level, and opportunities and some benefits of integration were outlined across the health system from policymaker to implementation and community levels. Participants also raised concerns over the feasibility of practical, workload and financial aspects of integration within existing health systems and HIV-specific issues to do with unintended harm from community-based HIV services that would need to be addressed proactively in any integrated platform.

### Achieving the ambitious UNAIDS 90–90–90 targets requires a strong community platform

Community platforms are likely to prove an essential component of HIV service expansion in Kenya in order to address the low uptake of services (particularly HIV testing services) among men, youth and couples.^27^ Providing services at home eliminates associated transport costs for clients, increases access and creates an environment that normalises HIV and HIV testing.^24^ A strong community platform would need to work outwards from a focus on coordination, competency, training and supervisory support systems at the health worker level to clarity on roles, tasks and remuneration at the policymaker level, as well as ensuring uptake and quality of service delivery at the community level. Should county governments choose to expand access to HIV services through community platforms there will also be a need for indicators to ensure equitable coverage and quality of services.^28^ Combining funding envelopes for decentralised HIV and existing community services is likely to increase the sustainability of these services. Furthermore, a strong community platform that includes HIV services will provide a governance and accountability framework for innovations like HIV self-testing that are currently being expanded in Kenyan communities to achieve UNAIDS targets.^29^

### Integrated approaches are desired at the community level and Kenya has a window of opportunity

The desire for a holistic approach to community-based care that includes HIV services and community-based testing is supported by integration at the community level that seeks to improve overall well-being rather than a particular condition.^18^ Integration also provides an opportunity for building on the pre-existing relationships with their communities that community health volunteers and community health extension workers enjoy by virtue of their unique interface between the community and the health system.^30^ Community engagement in selecting who is trained to provide HIV services, in promoting the programme and in sharing the content of training on confidentiality will play a vital part in building trusting relationships as highlighted in studies from Zambia and Kenya on community perceptions of home-based HIV testing approaches.^24^ ^31^ Community health workers visiting the homes of clients in other contexts have been shown to be effective in performing HIV testing and in delivering a range of other HIV services along the cascade such as adherence counselling and defaulter tracing.^6^ ^32–34^ These were paid and trained staff more akin to the community health extension workers than the community health volunteers in Kenya. A recent review of the literature on the evidence for integrating HIV and other health services, such as TB care, into national health systems, showed that the effect on health outcomes and quality of services was mixed.^35^ As Kenya moves forward with expanding HIV testing as a priority health service there is a need to seek synergies between vertical and horizontal programmes,^36^ ensuring that the integration of HIV services leads to a strengthening of community systems.^37^ ^38^

In Kenya community outreach has largely been provided by lay counsellors, but as external funding flat-lines, decisions need to be made on whether or not these lay counsellors are taken up into existing community structures and whether their tasks are shifted to existing community cadres or both.^39–41^ The current revision of the community health strategy and the process of devolution in Kenya provide a unique policy window for adopting a person-focused perspective that builds on current structures and expertise, provides a more coordinated approach between stakeholders and retains a highly skilled cadre of lay counsellors.^42^

### Feasibility and sustainability of integration

Participants highlighted multiple current and potential HIV-related roles for community providers, but the feasibility, appropriateness and sustainability of each role needs to be examined at each level of the system. Adding tasks to community health workers as part of scale-up can undermine the quality of services and divert scarce resources from priority interventions.^43^ ^44^ Findings from Ethiopia, where paid health extension workers have multiple tasks, reveal there is a need for clearly defined roles, standardised support, monitoring and accountability, strong referral links, supervision and training in order to realise benefits from the value of the interface role.^30^ For the integration of HIV services at community level to be feasible in Kenya it needs to be seen as a priority by communities and to use remunerated healthcare providers who are supported by strong policies and systems. Any proposed methods must take into account the community and the organisational settings.^45^ Integration will face constraints, such as the strain that financing newly converted lay counsellor-CHEWS may place on county budgets, and caution should be exercised in assuming that the current NGO-facilitated service landscape can necessarily be easily mapped onto government-run structures.

### Limitations

There are a number of limitations to our study. The study was carried out in only 2 of the 47 counties and both had been exposed to home-based testing and counselling services and a range of other HIV services provided by NGOs. The selection of community participants was carried out purposively by local administrators and home-based testing and counselling clients were selected by community health volunteers, thereby introducing a selection bias. Overall we had more female than male respondents and this was particularly marked among clients who had been tested, where more women were likely to be found at home. This gender bias may have influenced perceptions of the need for a holistic approach since women are the main beneficiaries of community health programmes that focus on maternal and child health. All of the lay counsellors interviewed were from a single NGO (that also employed the researchers) and had previously worked in the same counties. We used an anonymous online survey to avoid social desirability bias or power dynamics from the researchers being in the same institution. The lay counsellors would have all been exposed to the same way of working with CHVs and CHEWs in the community as it was agreed that CHVs would conduct community mobilisation, set up appointments and support the services, roles they did not necessarily perform in other counties or for other NGOs.

## Conclusion

HIV policymakers in Kenya are at a crossroads as they respond to a decline in funding for community programmes at the same time as increasing evidence indicates that they are effective and valued. The new community strategy provides a timely opportunity to incorporate and scale up HIV services into existing community health platforms in an equitable manner, an approach that appears to be well supported among study participants at all levels. Policymakers can take advantage of the opportunity of the new community strategy to incorporate and scale up HIV services in an equitable manner that is cognizant of potential constraints and challenges. For this to work, however, there is a need to address key health systems, funding and coordination issues at each level of the system: macro, meso and micro. One option would be to incorporate existing lay counsellors into the CHEW cadre and vice versa in line with national recommendations to formalise lay counsellors in the framework of the community strategy.^8^ Community health volunteers could then work alongside a capacitated community health extension worker cadre in support roles that increase uptake and outcomes of HIV services.^3^ ^6^
